# Editorial: Uterine fibroid surgery in gynecology and obstetrics and reproduction: lights and shadows

**DOI:** 10.3389/fsurg.2025.1698612

**Published:** 2025-09-25

**Authors:** Radmila Sparić, Safak Hatirnaz, Andrea Tinelli

**Affiliations:** 1 Faculty of Medicine University of Belgrade, Belgrade, Serbia; 2 Clinic for Gynecology and Obstetrics, University Clinical Center of Serbia, Belgrade, Serbia; 3 School of Health Sciences, Kent University, Istanbul, Türkiye; 4 Department of Obstetrics and Gynecology, CERICSAL (CEntro di RIcerca Clinico SALentino), “Veris Delli Ponti Hospital”, Scorrano, Italy

**Keywords:** fibroid, STUMP (smooth muscle tumours of uncertain malignant potential), cesarean myomectomy, surgery, artificial inteligence

The most prevalent benign tumors in women of reproductive age are uterine fibroids. The mainstay for treating symptomatic fibroids that don't respond to less invasive or medicinal treatments is still surgery. Myomectomy, which entails surgically removing fibroids while leaving the uterus intact, is the recommended course of action for women who want to preserve their fertility. Nevertheless, it doesn't stop new fibroids from growing (1–5). Women who have severe disease or no desire for conception may benefit from a hysterectomy, the final therapy that involves removing the uterus and resolves symptoms permanently. The benefit of a hysterectomy is that there is no subsequent recurrence of fibroids, despite the risks involved with major surgery (3, 6). Minimally invasive alternatives include uterine artery embolization (UAE), which deprives fibroids of their blood supply causing shrinkage but may carry pregnancy-related risks. Other noninvasive options such as magnetic resonance-guided focused ultrasound (MRgFUS) or high-intensity focused ultrasound (HIFU) offer thermal ablation of fibroids with symptom relief and uterine preservation. These modalities exhibit low complication rates and suit patients seeking uterus-sparing treatments (1, 3, 7).

Given the prevalence of uterine fibroids and the serious complications they can cause, this research topic consists of excellent, thought-provoking studies that are primarily focused on improving the diagnosis and treatment of these conditions. This work is essential and extremely valuable to both clinical practice and scientific research.

It has long been recognized that acupuncture exerts positive effects on human health and symptoms of various diseases, including uterine fibroids. Chen et al. demonstrated that traditional acupuncture and Chinese herbal medicine are more effective than herbal therapy alone; they reduce uterine and fibroid size, relieve symptoms, improve hormone balance, and improve general quality of life (8).

Enhanced recovery after surgery (ERAS) protocols aim to reduce perioperative discomfort and accelerate recovery. A clinical trial by Mao et al. applied acupuncture as an integral component of ERAS, finding it safe and well tolerated, effectively controlling visceral, incisional, and shoulder pain, and exerting anxiolytic effects when applied preoperatively. These benefits reduced opioid requirements and adverse effects, shortened hospitalization, and lowered treatment costs (9).

The clinical appearance and fibroid features dictate the unique indications for each therapy approach. Optimizing efficacy and safety requires careful monitoring of fibroid characteristics and how they respond to treatment. Xue et al. identified predictive factors for successful HIFU ablation, noting higher ablation rates—and thus lower resistance—in larger, highly echogenic, irregularly shaped fibroids, as well as those with attenuation bands, anterior wall lesions, or subserosal locations. Conversely, fibroids with necrotic foci exhibited greater resistance to ablation.

Furthermore, tracking fibroid pathogenesis-related biomarkers both before and after surgery is essential for assessing results and recurrence risk. The protocol proposed by Aimagambetova et al. explores correlations between angiogenic factors (e.g., VEGF), fibrotic growth factors (e.g., TGF-β), and clinical parameters such as menorrhagia score and quality of life following organ-preserving uterine artery embolization (10).

Cesarean myomectomy may present certain risks, potentially deterring clinicians. However, research by Güler et al. indicates that cesarean myomectomy, performed via either transendometrial or serosal approaches, does not significantly differ in obstetric, perinatal, or surgical outcomes compared to cesarean section alone. This supports its safety and efficacy as a durable management option for fibroids at delivery.

The advent of new technologies significantly enhances fibroid diagnosis and decision-making by reducing subjectivity. Chen et al. introduced an AI algorithm based on machine and deep learning that improves MRI interpretation objectivity, procedural planning, reduces hysteroscopy duration, intraoperative blood loss, and accelerates postoperative recovery.

Five fascinating case studies that highlight uncommon and difficult clinical situations are included in this edition. There are significant risks for both the mother and the fetus in a twin pregnancy after laparoscopic adenomyomectomy, such as placenta accreta spectrum and uterine rupture. With careful patient screening, Ota et al. reported a successful dichorionic diamniotic twin pregnancy delivered by elective cesarean at 32 weeks.

Dong et al. presented a rare and severe case of a giant 27 cm fibroid causing prolapse through the cervix, deep venous thrombosis, pulmonary embolism, cardiac and renal failure, and shock. Following inferior vena cava filter placement and extensive hysterectomy, the patient fully recovered.

Bićanin-Ilić et al. described a smooth muscle tumor of uncertain malignant potential (STUMP) progressing rapidly to high-grade leiomyosarcoma with uterine rupture, hemoperitoneum, and widespread metastases, highlighting risks of malignant transformation.

Huang et al. reported a case initially diagnosed as a benign leiomyoma that was later identified as endometrial stromal sarcoma with mixed grades and a rare mutation, underscoring how malignancies may masquerade as benign lesions; metastases were found in the pelvis and inferior vena cava.

Hu et al. encountered a rare benign metastasizing leiomyoma with metastases to the anterior abdominal wall involving fascia and muscle, illustrating that histologically benign tumors can sometimes display unusual metastatic patterns.

The collection of material included in this issue shows how uterine fibroids are being managed, from non-invasive, holistic approaches to innovative therapeutics and careful oncologic evaluation. Even though there is a lot of hope due to advancements, problems including imprecise diagnosis, risky surgery, and possible malignant transformation still exist. Future studies must keep improving tailored strategies that strike a compromise between safety, efficacy, preserving fertility, and incorporating new technology.
